# Methyl 2,3-di-*O*-acetyl-4-*O*-levulinoyl-1-*O*-(2,2,2-trichloro-2-imino­ethyl)-l-ido­pyran­osiduronate

**DOI:** 10.1107/S1600536810010895

**Published:** 2010-03-27

**Authors:** Chao Cai, Guohua Wei, Yuguo Du

**Affiliations:** a Research Center for Eco-Environmental Sciences, Chinese Academy of Sciences, Beijing 100085, People’s Republic of China

## Abstract

In the title compound, C_18_H_22_Cl_3_NO_11_, a novel derivative of l-idopyran­osiduronic acid, the six-membered ring adopts a chair conformation.

## Related literature

For background to l-iduronic acids, see: Capila & Linhardt (2002 ▶); Jobron & Jacquinet (1998 ▶); Lee *et al.* (2004 ▶). For the synthesis of iduronic acid derivatives, see: Yu *et al.* (2004 ▶); Sanjoy *et al.* (2001 ▶); Lubineau *et al.* (2000 ▶); Lohman *et al.* (2003 ▶).
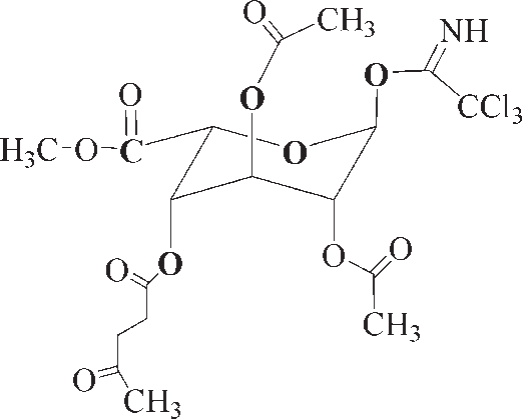


## Experimental

### 

#### Crystal data

C_18_H_22_Cl_3_NO_11_
*M* *_r_* = 534.72
Orthorhombic, 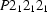

*a* = 9.0498 (10) Å
*b* = 9.7560 (11) Å
*c* = 26.570 (3) Å
*V* = 2345.8 (4) Å^3^
*Z* = 4
Cu *K*α radiation
μ = 4.07 mm^−1^
*T* = 173 K
0.41 × 0.30 × 0.28 mm

#### Data collection

Rigaku R-AXIS RAPID IP area-detector diffractometer
Absorption correction: numerical (*ABSCOR*; Higashi, 1995 ▶) *T*_min_ = 0.286, *T*_max_ = 0.395
16340 measured reflections
4259 independent reflections
3893 reflections with *I* > 2σ(*I*)
*R*_int_ = 0.047

#### Refinement

*R*[*F* ^2^ > 2σ(*F* ^2^)] = 0.046
*wR*(*F* ^2^) = 0.100
*S* = 1.09
4259 reflections
302 parameters
H-atom parameters constrained
Δρ_max_ = 0.45 e Å^−3^
Δρ_min_ = −0.48 e Å^−3^
Absolute structure: Flack (1983 ▶), 1804 Friedel pairs
Flack parameter: 0.023 (18)

### 

Data collection: *RAPID-AUTO* (Rigaku, 2001 ▶); cell refinement: *RAPID-AUTO*; data reduction: *RAPID-AUTO*; program(s) used to solve structure: *SHELXS97* (Sheldrick, 2008 ▶); program(s) used to refine structure: *SHELXL97* (Sheldrick, 2008 ▶); molecular graphics: *XP* (Sheldrick, 2008 ▶); software used to prepare material for publication: *SHELXL97*.

## Supplementary Material

Crystal structure: contains datablocks I, global. DOI: 10.1107/S1600536810010895/bt5207sup1.cif

Structure factors: contains datablocks I. DOI: 10.1107/S1600536810010895/bt5207Isup2.hkl

Additional supplementary materials:  crystallographic information; 3D view; checkCIF report

## Figures and Tables

**Fig. 1.**
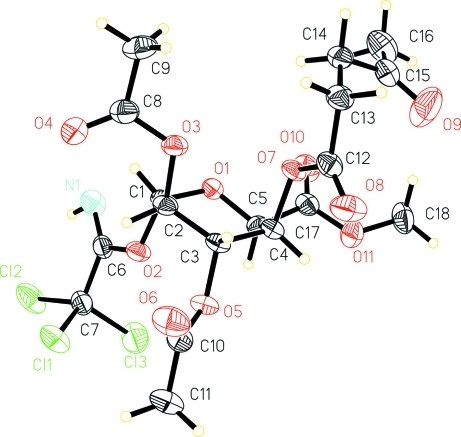
The molecular structure of the title compound, displacement ellipsoids are at the 50% level.

**Fig. 2.**
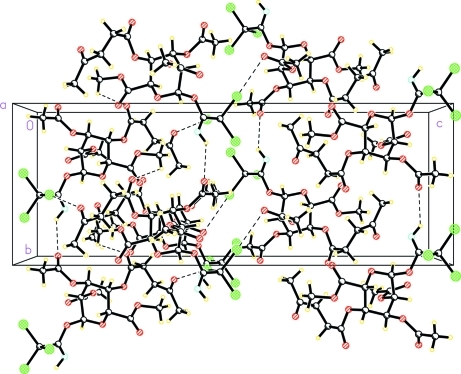
A packing diagram of the title compound.

## supplementary crystallographic information

### Comment

L-iduronic acids are key components of numerous biologically potent oligosaccharides and glycopeptides (Capila & Linhardt, 2002). For example, heparin, heparan sulfate (Jobron & Jacquinet, 1998), dermatan sulfate (Lee *et al.*, 2004). This series of glycosaminoglycans plays an important role in a diverse set of biological processes which all contain L-idopyranosiduronic acids. To study the structure-activity relationship of such polymers, there is a need for chemically pure oligosaccharide sequences which can be prepared by organic syntheses.

Since iduronic acid itself is not commercially available, syntheses of iduronic acid derivatives (Yu *et al.*, 2004) from a variety of starting materials, including idose, glucose, glycals,and glucuronic acid have been developed (Lubineau *et al.*, 2000 & Lohman *et al.*, 2003). Herein, we have explored a novel and efficient route toward the synthesis of L-idopyranosiduronate trichloroacetimidate which will be used as a key building block to synthesize dermatan sulfate.

### Experimental

The title compound was prepared in 76.8% yield by treatment with trichloroaceonitrile and DBU at 0 °C. The reaction was stirred for 30 min then allowed to warm to room temperature. Solvent was evaporated and the residue purified by Flash silica gel column chromatography (silica quenched with 1% NEt_3_) afford the title compound as syrupy.

### Refinement

H atoms were positioned geometrically and allowed to ride on their parent atoms, with C—H = 0.95—1.00 Å and U_iso_(H) = 1.2—1.5U_eq_(C).

### Crystal data

**Table:**

C_18_H_22_Cl_3_NO_11_	*D*_x_ = 1.514 Mg m^−^^3^
*M**_r_* = 534.72	Cu *K*α radiation, λ = 1.54186 Å
Orthorhombic, *P*2_1_2_1_2_1_	Cell parameters from 1098 reflections
*a* = 9.0498 (10) Å	θ = 2.2–27.5°
*b* = 9.7560 (11) Å	µ = 4.07 mm^−^^1^
*c* = 26.570 (3) Å	*T* = 173 K
*V* = 2345.8 (4) Å^3^	Block, colorless
*Z* = 4	0.41 × 0.30 × 0.28 mm
*F*(000) = 1104	

### Data collection

**Table:**

Rigaku R-AXIS RAPID IP area-detector diffractometer	4259 independent reflections
Radiation source: rotating anode	3893 reflections with *I* > 2σ(*I*)
graphite	*R*_int_ = 0.047
ω scans at fixed χ = 45°	θ_max_ = 68.2°, θ_min_ = 3.3°
Absorption correction: numerical (*ABSCOR*; Higashi, 1995)	*h* = −10→10
*T*_min_ = 0.286, *T*_max_ = 0.395	*k* = −11→11
16340 measured reflections	*l* = −32→31

### Refinement

**Table:**

Refinement on *F*^2^	Secondary atom site location: difference Fourier map
Least-squares matrix: full	Hydrogen site location: inferred from neighbouring sites
*R*[*F*^2^ > 2σ(*F*^2^)] = 0.046	H-atom parameters constrained
*wR*(*F*^2^) = 0.100	*w* = 1/[σ^2^(*F*_o_^2^) + (0.0268*P*)^2^ + 1.8534*P*] where *P* = (*F*_o_^2^ + 2*F*_c_^2^)/3
*S* = 1.09	(Δ/σ)_max_ < 0.001
4259 reflections	Δρ_max_ = 0.45 e Å^−^^3^
302 parameters	Δρ_min_ = −0.48 e Å^−^^3^
0 restraints	Absolute structure: Flack (1983), 1804 Friedel pairs
Primary atom site location: structure-invariant direct methods	Flack parameter: 0.023 (18)

### Special details

**Table:**

Geometry. All esds (except the esd in the dihedral angle between two l.s. planes) are estimated using the full covariance matrix. The cell esds are taken into account individually in the estimation of esds in distances, angles and torsion angles; correlations between esds in cell parameters are only used when they are defined by crystal symmetry. An approximate (isotropic) treatment of cell esds is used for estimating esds involving l.s. planes.
Refinement. Refinement of *F*^2^ against ALL reflections. The weighted *R*-factor wR and goodness of fit *S* are based on *F*^2^, conventional *R*-factors *R* are based on *F*, with *F* set to zero for negative *F*^2^. The threshold expression of *F*^2^ > σ(*F*^2^) is used only for calculating *R*-factors(gt) etc. and is not relevant to the choice of reflections for refinement. *R*-factors based on *F*^2^ are statistically about twice as large as those based on *F*, and *R*- factors based on ALL data will be even larger.

### Fractional atomic coordinates and isotropic or equivalent isotropic displacement parameters (Å^2^)

**Table:**

	*x*	*y*	*z*	*U*_iso_*/*U*_eq_	
Cl1	0.43622 (11)	0.42769 (10)	−0.00915 (3)	0.0470 (2)	
Cl2	0.44529 (14)	0.72085 (10)	−0.00601 (4)	0.0578 (3)	
Cl3	0.62102 (10)	0.56765 (16)	0.06361 (4)	0.0664 (3)	
O1	0.2587 (2)	0.4459 (2)	0.17647 (7)	0.0275 (5)	
O2	0.3250 (2)	0.4443 (2)	0.09152 (8)	0.0329 (5)	
O3	0.0181 (2)	0.2548 (2)	0.14357 (8)	0.0332 (5)	
O4	−0.0977 (3)	0.3313 (3)	0.07477 (9)	0.0527 (7)	
O5	0.3933 (2)	0.1578 (2)	0.10987 (8)	0.0320 (5)	
O6	0.2917 (3)	−0.0176 (3)	0.06774 (10)	0.0488 (7)	
O7	0.1922 (2)	0.1967 (2)	0.22751 (8)	0.0285 (5)	
O8	0.2316 (3)	−0.0295 (2)	0.23558 (11)	0.0493 (7)	
O9	0.1750 (4)	0.1508 (4)	0.35252 (12)	0.0780 (11)	
O10	0.3492 (3)	0.4899 (3)	0.27135 (9)	0.0462 (7)	
O11	0.4850 (3)	0.2966 (3)	0.26989 (8)	0.0402 (6)	
N1	0.2424 (3)	0.6658 (3)	0.08223 (11)	0.0411 (7)	
H1A	0.2761	0.7351	0.0630	0.049*	
C1	0.2099 (3)	0.4196 (4)	0.12743 (10)	0.0284 (7)	
H1C	0.1257	0.4827	0.1198	0.034*	
C2	0.1588 (3)	0.2725 (3)	0.11851 (12)	0.0280 (7)	
H2A	0.1470	0.2558	0.0816	0.034*	
C3	0.2619 (3)	0.1663 (3)	0.14075 (11)	0.0269 (7)	
H3A	0.2117	0.0751	0.1419	0.032*	
C4	0.3162 (4)	0.2048 (3)	0.19295 (11)	0.0260 (7)	
H4A	0.3971	0.1416	0.2038	0.031*	
C5	0.3679 (4)	0.3526 (3)	0.19531 (11)	0.0271 (7)	
H5A	0.4609	0.3628	0.1754	0.033*	
C6	0.3286 (4)	0.5705 (4)	0.06991 (11)	0.0315 (7)	
C7	0.4526 (4)	0.5722 (4)	0.03110 (11)	0.0352 (7)	
C8	−0.1037 (4)	0.2905 (4)	0.11727 (13)	0.0366 (8)	
C9	−0.2403 (4)	0.2721 (4)	0.14798 (15)	0.0458 (10)	
H9A	−0.3271	0.2931	0.1273	0.069*	
H9B	−0.2373	0.3341	0.1770	0.069*	
H9C	−0.2461	0.1771	0.1598	0.069*	
C10	0.3914 (4)	0.0610 (4)	0.07321 (11)	0.0343 (7)	
C11	0.5308 (4)	0.0652 (4)	0.04323 (13)	0.0471 (9)	
H11A	0.5075	0.0543	0.0074	0.071*	
H11B	0.5962	−0.0092	0.0540	0.071*	
H11C	0.5802	0.1535	0.0485	0.071*	
C12	0.1578 (4)	0.0694 (4)	0.24437 (11)	0.0323 (7)	
C13	0.0114 (4)	0.0687 (4)	0.27120 (12)	0.0350 (7)	
H13A	0.0108	−0.0084	0.2954	0.042*	
H13B	−0.0674	0.0511	0.2462	0.042*	
C14	−0.0258 (4)	0.1992 (4)	0.29934 (12)	0.0349 (8)	
H14A	−0.0291	0.2762	0.2751	0.042*	
H14B	−0.1254	0.1898	0.3143	0.042*	
C15	0.0820 (4)	0.2329 (4)	0.34006 (13)	0.0434 (9)	
C16	0.0726 (6)	0.3724 (4)	0.36322 (14)	0.0572 (12)	
H16A	0.1519	0.3832	0.3881	0.086*	
H16B	−0.0233	0.3832	0.3799	0.086*	
H16C	0.0832	0.4422	0.3369	0.086*	
C17	0.3974 (4)	0.3918 (3)	0.24983 (12)	0.0308 (7)	
C18	0.5206 (4)	0.3098 (4)	0.32281 (13)	0.0481 (10)	
H18A	0.5860	0.2346	0.3329	0.072*	
H18B	0.4295	0.3062	0.3427	0.072*	
H18C	0.5705	0.3975	0.3286	0.072*	

### Atomic displacement parameters (Å^2^)

**Table:**

	*U*^11^	*U*^22^	*U*^33^	*U*^12^	*U*^13^	*U*^23^
Cl1	0.0627 (6)	0.0425 (5)	0.0358 (4)	−0.0035 (5)	0.0135 (4)	−0.0068 (4)
Cl2	0.0917 (8)	0.0412 (5)	0.0405 (5)	−0.0004 (5)	0.0219 (5)	0.0099 (4)
Cl3	0.0339 (5)	0.1146 (10)	0.0507 (5)	−0.0158 (6)	−0.0041 (4)	0.0145 (7)
O1	0.0301 (11)	0.0272 (12)	0.0252 (10)	0.0002 (10)	0.0028 (9)	−0.0002 (9)
O2	0.0336 (12)	0.0322 (13)	0.0330 (11)	0.0029 (11)	0.0122 (10)	0.0045 (10)
O3	0.0228 (12)	0.0440 (14)	0.0328 (11)	−0.0007 (11)	0.0049 (10)	−0.0025 (10)
O4	0.0407 (15)	0.079 (2)	0.0381 (15)	0.0149 (15)	−0.0022 (12)	−0.0001 (14)
O5	0.0287 (12)	0.0353 (13)	0.0321 (11)	−0.0007 (10)	0.0113 (10)	−0.0070 (10)
O6	0.0489 (16)	0.0484 (16)	0.0492 (15)	−0.0084 (14)	0.0079 (13)	−0.0188 (13)
O7	0.0303 (12)	0.0276 (12)	0.0276 (11)	−0.0022 (10)	0.0105 (10)	0.0035 (9)
O8	0.0546 (17)	0.0269 (14)	0.0664 (18)	0.0040 (13)	0.0261 (15)	0.0048 (12)
O9	0.079 (2)	0.089 (3)	0.067 (2)	0.029 (2)	−0.0329 (19)	−0.0091 (19)
O10	0.0583 (17)	0.0445 (16)	0.0359 (13)	0.0033 (14)	−0.0002 (13)	−0.0117 (12)
O11	0.0410 (14)	0.0437 (14)	0.0358 (12)	0.0008 (12)	−0.0087 (11)	0.0005 (11)
N1	0.052 (2)	0.0306 (16)	0.0402 (16)	0.0008 (16)	0.0072 (15)	0.0049 (13)
C1	0.0252 (15)	0.0342 (17)	0.0259 (14)	−0.0011 (15)	0.0053 (12)	0.0028 (15)
C2	0.0214 (15)	0.0346 (18)	0.0281 (16)	−0.0045 (15)	0.0052 (13)	−0.0032 (14)
C3	0.0249 (16)	0.0300 (17)	0.0258 (15)	−0.0021 (14)	0.0094 (13)	−0.0001 (13)
C4	0.0266 (16)	0.0278 (17)	0.0236 (15)	−0.0022 (14)	0.0063 (13)	−0.0010 (13)
C5	0.0237 (16)	0.0273 (16)	0.0304 (16)	−0.0014 (14)	0.0025 (14)	0.0014 (13)
C6	0.0374 (17)	0.0334 (18)	0.0237 (15)	0.0014 (17)	−0.0004 (13)	0.0029 (15)
C7	0.0366 (18)	0.0417 (19)	0.0273 (15)	−0.0030 (18)	−0.0002 (14)	0.0004 (15)
C8	0.0291 (18)	0.043 (2)	0.0373 (19)	−0.0020 (17)	0.0018 (15)	−0.0103 (16)
C9	0.0248 (19)	0.055 (2)	0.058 (2)	−0.0024 (18)	0.0058 (17)	−0.006 (2)
C10	0.0381 (18)	0.0355 (18)	0.0295 (16)	0.0043 (18)	0.0005 (14)	−0.0050 (15)
C11	0.050 (2)	0.050 (2)	0.0415 (19)	0.002 (2)	0.0182 (17)	−0.0085 (19)
C12	0.0360 (18)	0.0289 (17)	0.0319 (16)	−0.0055 (17)	0.0062 (14)	0.0035 (15)
C13	0.0318 (17)	0.0350 (18)	0.0383 (17)	−0.0042 (17)	0.0068 (14)	0.0049 (16)
C14	0.0305 (18)	0.0380 (19)	0.0363 (17)	0.0014 (16)	0.0072 (15)	0.0054 (15)
C15	0.048 (2)	0.055 (2)	0.0276 (17)	0.002 (2)	0.0040 (17)	0.0029 (17)
C16	0.075 (3)	0.058 (3)	0.039 (2)	−0.008 (2)	0.007 (2)	−0.0088 (18)
C17	0.0275 (16)	0.0347 (19)	0.0301 (16)	−0.0089 (15)	0.0022 (14)	−0.0008 (14)
C18	0.047 (2)	0.065 (3)	0.0320 (18)	−0.011 (2)	−0.0091 (17)	0.0113 (18)

### Geometric parameters (Å, °)

**Table:**

Cl1—C7	1.776 (4)	C4—C5	1.517 (4)
Cl2—C7	1.754 (4)	C4—H4A	1.0000
Cl3—C7	1.753 (3)	C5—C17	1.522 (4)
O1—C1	1.399 (3)	C5—H5A	1.0000
O1—C5	1.433 (4)	C6—C7	1.524 (4)
O2—C6	1.359 (4)	C8—C9	1.492 (5)
O2—C1	1.433 (3)	C9—H9A	0.9800
O3—C8	1.351 (4)	C9—H9B	0.9800
O3—C2	1.447 (3)	C9—H9C	0.9800
O4—C8	1.199 (4)	C10—C11	1.492 (4)
O5—C10	1.357 (4)	C11—H11A	0.9800
O5—C3	1.447 (3)	C11—H11B	0.9800
O6—C10	1.194 (4)	C11—H11C	0.9800
O7—C12	1.356 (4)	C12—C13	1.504 (4)
O7—C4	1.452 (3)	C13—C14	1.515 (5)
O8—C12	1.197 (4)	C13—H13A	0.9900
O9—C15	1.208 (5)	C13—H13B	0.9900
O10—C17	1.197 (4)	C14—C15	1.493 (5)
O11—C17	1.333 (4)	C14—H14A	0.9900
O11—C18	1.448 (4)	C14—H14B	0.9900
N1—C6	1.256 (4)	C15—C16	1.496 (5)
N1—H1A	0.9001	C16—H16A	0.9800
C1—C2	1.527 (4)	C16—H16B	0.9800
C1—H1C	1.0000	C16—H16C	0.9800
C2—C3	1.514 (4)	C18—H18A	0.9800
C2—H2A	1.0000	C18—H18B	0.9800
C3—C4	1.518 (4)	C18—H18C	0.9800
C3—H3A	1.0000		
			
C1—O1—C5	115.3 (2)	C8—C9—H9A	109.5
C6—O2—C1	116.8 (2)	C8—C9—H9B	109.5
C8—O3—C2	116.7 (2)	H9A—C9—H9B	109.5
C10—O5—C3	115.9 (2)	C8—C9—H9C	109.5
C12—O7—C4	115.9 (2)	H9A—C9—H9C	109.5
C17—O11—C18	117.3 (3)	H9B—C9—H9C	109.5
C6—N1—H1A	101.4	O6—C10—O5	123.0 (3)
O1—C1—O2	111.1 (2)	O6—C10—C11	126.3 (3)
O1—C1—C2	114.3 (3)	O5—C10—C11	110.7 (3)
O2—C1—C2	105.9 (2)	C10—C11—H11A	109.5
O1—C1—H1C	108.4	C10—C11—H11B	109.5
O2—C1—H1C	108.4	H11A—C11—H11B	109.5
C2—C1—H1C	108.4	C10—C11—H11C	109.5
O3—C2—C3	106.3 (2)	H11A—C11—H11C	109.5
O3—C2—C1	107.9 (2)	H11B—C11—H11C	109.5
C3—C2—C1	113.3 (3)	O8—C12—O7	123.1 (3)
O3—C2—H2A	109.7	O8—C12—C13	125.5 (3)
C3—C2—H2A	109.7	O7—C12—C13	111.3 (3)
C1—C2—H2A	109.7	C12—C13—C14	115.2 (3)
O5—C3—C2	108.9 (2)	C12—C13—H13A	108.5
O5—C3—C4	105.4 (2)	C14—C13—H13A	108.5
C2—C3—C4	112.8 (3)	C12—C13—H13B	108.5
O5—C3—H3A	109.9	C14—C13—H13B	108.5
C2—C3—H3A	109.9	H13A—C13—H13B	107.5
C4—C3—H3A	109.9	C15—C14—C13	113.4 (3)
O7—C4—C5	105.3 (2)	C15—C14—H14A	108.9
O7—C4—C3	108.3 (2)	C13—C14—H14A	108.9
C5—C4—C3	111.9 (3)	C15—C14—H14B	108.9
O7—C4—H4A	110.4	C13—C14—H14B	108.9
C5—C4—H4A	110.4	H14A—C14—H14B	107.7
C3—C4—H4A	110.4	O9—C15—C14	120.5 (4)
O1—C5—C4	112.1 (3)	O9—C15—C16	122.0 (4)
O1—C5—C17	107.1 (3)	C14—C15—C16	117.5 (4)
C4—C5—C17	109.4 (3)	C15—C16—H16A	109.5
O1—C5—H5A	109.4	C15—C16—H16B	109.5
C4—C5—H5A	109.4	H16A—C16—H16B	109.5
C17—C5—H5A	109.4	C15—C16—H16C	109.5
N1—C6—O2	123.1 (3)	H16A—C16—H16C	109.5
N1—C6—C7	128.7 (3)	H16B—C16—H16C	109.5
O2—C6—C7	108.2 (3)	O10—C17—O11	125.6 (3)
C6—C7—Cl3	107.9 (2)	O10—C17—C5	126.3 (3)
C6—C7—Cl2	111.2 (3)	O11—C17—C5	108.1 (3)
Cl3—C7—Cl2	109.31 (19)	O11—C18—H18A	109.5
C6—C7—Cl1	109.7 (2)	O11—C18—H18B	109.5
Cl3—C7—Cl1	110.4 (2)	H18A—C18—H18B	109.5
Cl2—C7—Cl1	108.34 (16)	O11—C18—H18C	109.5
O4—C8—O3	122.4 (3)	H18A—C18—H18C	109.5
O4—C8—C9	126.3 (4)	H18B—C18—H18C	109.5
O3—C8—C9	111.2 (3)		
			
C5—O1—C1—O2	−68.1 (3)	C3—C4—C5—C17	171.0 (3)
C5—O1—C1—C2	51.8 (3)	C1—O2—C6—N1	4.3 (5)
C6—O2—C1—O1	−94.0 (3)	C1—O2—C6—C7	−176.8 (2)
C6—O2—C1—C2	141.3 (3)	N1—C6—C7—Cl3	108.7 (4)
C8—O3—C2—C3	−151.7 (3)	O2—C6—C7—Cl3	−70.2 (3)
C8—O3—C2—C1	86.4 (3)	N1—C6—C7—Cl2	−11.1 (5)
O1—C1—C2—O3	72.9 (3)	O2—C6—C7—Cl2	170.0 (2)
O2—C1—C2—O3	−164.5 (2)	N1—C6—C7—Cl1	−131.0 (3)
O1—C1—C2—C3	−44.6 (3)	O2—C6—C7—Cl1	50.2 (3)
O2—C1—C2—C3	78.1 (3)	C2—O3—C8—O4	2.0 (5)
C10—O5—C3—C2	−93.1 (3)	C2—O3—C8—C9	−177.9 (3)
C10—O5—C3—C4	145.6 (3)	C3—O5—C10—O6	−3.6 (5)
O3—C2—C3—O5	167.4 (2)	C3—O5—C10—C11	178.4 (3)
C1—C2—C3—O5	−74.3 (3)	C4—O7—C12—O8	6.8 (5)
O3—C2—C3—C4	−75.9 (3)	C4—O7—C12—C13	−168.6 (3)
C1—C2—C3—C4	42.4 (3)	O8—C12—C13—C14	152.1 (4)
C12—O7—C4—C5	−159.4 (3)	O7—C12—C13—C14	−32.6 (4)
C12—O7—C4—C3	80.8 (3)	C12—C13—C14—C15	−61.0 (4)
O5—C3—C4—O7	−172.2 (2)	C13—C14—C15—O9	−9.9 (5)
C2—C3—C4—O7	69.0 (3)	C13—C14—C15—C16	168.3 (3)
O5—C3—C4—C5	72.1 (3)	C18—O11—C17—O10	2.6 (5)
C2—C3—C4—C5	−46.6 (3)	C18—O11—C17—C5	−176.6 (3)
C1—O1—C5—C4	−55.9 (3)	O1—C5—C17—O10	−6.6 (4)
C1—O1—C5—C17	−175.8 (2)	C4—C5—C17—O10	−128.3 (4)
O7—C4—C5—O1	−65.1 (3)	O1—C5—C17—O11	172.7 (2)
C3—C4—C5—O1	52.4 (3)	C4—C5—C17—O11	50.9 (3)
O7—C4—C5—C17	53.6 (3)		
